# Small is beautiful–a glycolytic metabolite signals mTORC1 activation in cancer cell metabolism

**DOI:** 10.1038/s41392-020-00371-9

**Published:** 2020-11-03

**Authors:** Angela M. Otto

**Affiliations:** Munich Institute of BioEngineering, Technical University of Munich, 85748 Garching, Germany

**Keywords:** Biochemistry, Cancer metabolism

Even though the mTOR complex has been known for decades as a nutrient sensor, being thus in focus for drug targeting in metabolic disease and cancer metabolism, it has remained puzzling how it is able to get a sense of glucose availability in cells. Now, a small metabolite of the glycolytic pathway, dihydroxyacetone phosphate, has been identified by Sabatini and his coworkers, who show how mTORC1-activity is regulated in glucose-starved and replenished cancer cells.^1^

In a microenvironment of normal tissues and cancers, nutrient fluctuations are inevitable, a condition to which cells must quickly adapt their energy metabolism—without requiring translational and transcriptional activities. A classical sensor of the energy status is AMP-activated kinase (AMPK), which is activated by high AMP/ATP ratios and stimulates glucose metabolism. Another is mTORC1, which is also known as a regulator for amino acid and lipid metabolism.^2,3^ In spite of its interaction with transcription factors, for example, HIF1 and c-myc, whose expression can indirectly regulate glucose metabolism in cancer cells,^4^ there has been no consensus on how the dynamics of glucose metabolism regulate mTORC1 activation; both AMPK-dependent and independent pathways have been postulated.^2^

**Fig. 1:**
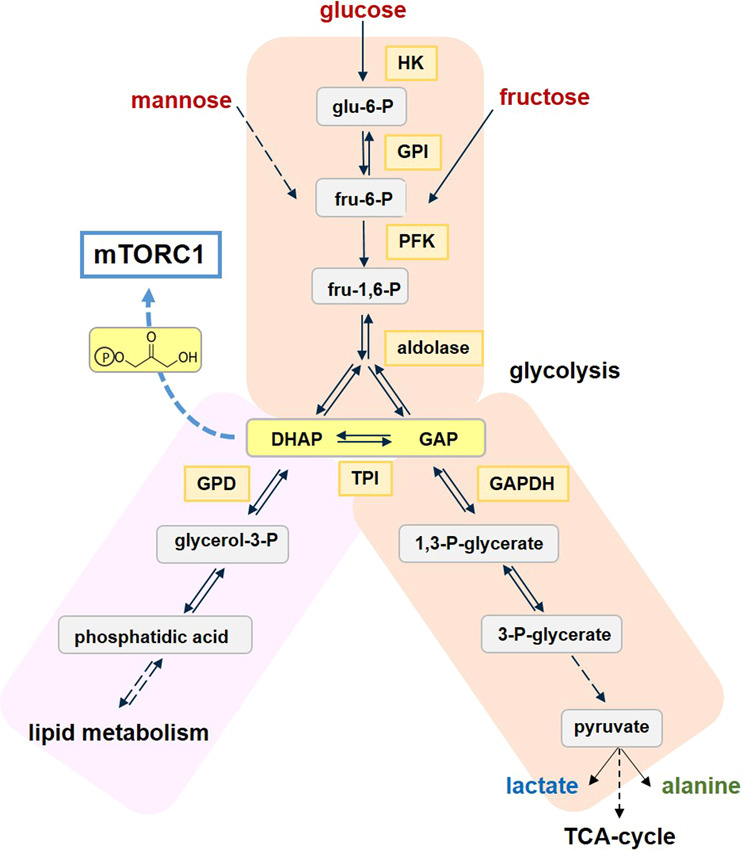
The metabolic home of a sugar-derived metabolite signaling to mTORC1. Glycolysis following uptake of glucose, fructose, and mannose proceeds to the trioses dihydroxyacetone phosphate (DHAP) and glyceraldehyde-3-phosphate (GAP), which are at a metabolic junction between further glycolytic reactions and lipid synthesis. Those enzymes knocked out or inhibited in the presented study (Orozco et al.^1^) are highlighted. HK hexokinase; GPI glucose-6-phosphate isomerase; PFK phosphofructokinase; TPI triose phosphate isomerase; GDP glycerol-3-phosphate dehydrogenase; GAPDH glyceraldehyde-3-phophate dehydrogenase. Dashed lines indicate that several steps are involved; in the case of mTORC1 these are yet unknown

What could be a metabolic component for translating glucose supply into signals regulating metabolism? To find a direct link to mTORC1-based regulation, one that is AMPK-independent, Sabatini and coworkers^1^ developed a genetically engineered tumor cell system, in which the expression of AMPK was knocked out and the cells had to rely on mTORC1 for glucose sensing. When such cells were starved for glucose, mTORC1 activity, measured as phosphorylation of S6-kinase, was almost undetectable, while the addition of glucose at physiologically low levels reactivated mTORC1. Using the principle of this setup, the contribution of sequential glycolytic reactions was dissected by conditionally knocking out or inhibiting specific enzymes one by one (Fig. 1).

Upon cellular uptake of sugars, the glycolytic process is initiated by hexokinase (HK), a rate-limiting enzyme, which phosphorylates glucose as well as fructose, but not mannose. Nevertheless, mTORC1 was activated by all three sugars when served to glucose-starved cells; this confirms that mTORC1 can act as a sugar sensor independently of AMPK and is stimulated not only by glucose. Next, knocking out glucose-6-phosphate isomerase (GPI), thereby interrupting the formation of fructose-6-phosphate, did not allow glucose to activate mTORC1—in contrast to mannose; this indicated that this phosphorylated intermediate is required. When knocking out aldolase, again there was no activation of mTORC1, and metabolite profiling revealed very low levels of its downstream metabolites dihydroxyacetone phosphate (DHAP) and glyceraldehyde phosphate (GAP), but also of phosphoenol pyruvate (PEP).^1^ It is noteworthy that phosphofructokinase (PFK), providing the substrate for aldolase, is a key rate-limiting enzyme in glycolysis; but neither its substrate nor an increase in its product fructose-1,6-phosphate affected mTORC1 activity.

In the lower part of glycolysis, a key enzyme is glyceraldehyde dehydrogenase (GAPDH) (Fig. 1), oxidizing GAP to 1,3-bisphosphate glycerate while converting NAD^+^ to NADH. When its activity was inhibited by koningic acid at the onset of glucose starvation, mTORC1 remained activated for some time, while the levels of upstream metabolites increased and those of downstream metabolites decreased. On the other hand, after prolonged glucose-starvation, feeding the cells briefly with glucose in the presence of the GAPDH inhibitor did not reactivate mTORC1 activity, due to depleted metabolite levels. Together, these and further results converged on DHAP and/or GAP being potential sensor molecules for mTORC1 activation.

To identify which of these two metabolites might be the sensor, the catalyzed interconversion of DHAP <-> GAP was blocked by knocking out triose-phosphate isomerase (TPI) (Fig. 1). When such cells were depleted of glucose, their initially high level of DHAP gradually declined, and this was accompanied by reduced mTORC1 activity. This further supports DHAP being a sugar sensor.

However, mTORC1 is also involved in lipid metabolism,^3^ and DHAP is a precursor of lipid synthesis (Fig. 1). Could a related metabolite (also) be a potential sensor? In the first step, DHAP is converted to glycerol-3-phosphate (G3P) by G3P dehydrogenase (GPD). When this enzyme was overexpressed, glucose-stimulated mTORC1 activation was reduced, which can be explained by enhanced DHAP consumption. On the other hand, artificially enhancing DHAP levels in conditions of glucose starvation, resulted in mTORC1 activation. Moreover, the fact that the cellular levels of both DHAP and GAP, which are basically quite low, fluctuated about 10-fold between starvation and satiating glucose conditions, underlines their sensitive role in metabolic regulation. Thus, DHAP, independent of its metabolic origin, is responsible for signaling to mTORC1, with GAP probably having an auxiliary role.

This raises the question: are upstream components of the signaling pathway regulating mTORC1 activation involved? Such regulatory components could lead to changes in lysosomal localization and interactions of the mTOR complex with other regulatory factors.^4^ Rag-GTPases are responsible for mTORC1 recruitment to lysosomes in amino acid sensing. The present study^1^ shows that the loss Rag GTPase-associated proteins, namely GATOR1 (with KICSTOR) and GATOR2, abrogated glucose-sensitivity for mTORC1 activity, namely, by suppressing mTOR inhibition upon glucose starvation and by preventing its reactivation upon glucose supply, respectively. This indicates that glucose sensitivity of mTORC1 requires components binding to the lysosomal surface. As yet, a sensor protein for DHAP could not be identified, and there are no indications that glycolytic enzymes themselves are directly involved.^1^

The authors of the paper^1^ stress that their findings are an example of how mTORC1 regulates glucose metabolism in this particular cellular model system. The regulation may well be different in other cell types, which have a different metabolic program. Yet, this study provides a layout for developing further concepts and investigations on metabolic regulation by mTORC1 in other cell systems.

The concept that DHAP serves as a nutrient sensor for mTORC1, which is independent of the energy-sensor AMPK, is sensitive to glucose and at the junction to lipid metabolism, opens new perspectives: for therapeutic targeting associated with mTOR signaling, for treatment of drugs resistance, and for dietary inventions in the clinic.^2,4,5^
